# Magnitude of neonatal asphyxia and its predictors among newborns at public hospitals of Wolaita Zone in Southern Ethiopia, 2023

**DOI:** 10.1186/s12887-024-04627-z

**Published:** 2024-02-27

**Authors:** Shewazerf Gizachew, Girma Wogie, Mekasha Getnet, Arega Abebe Lonsako

**Affiliations:** 1 Department of Nursing, College of Medicine and Health Science, Wachemo University, Hossaina, Ethiopia; 2 School of Nursing and Midwifery, College of Medicine and Health Science, Debre Berhan University, Debre Berhan, Ethiopia; 3 School of Nursing, College of Medicine and Health Science, Arba Minch University, Arba Minch, Ethiopia

**Keywords:** Neonatal asphyxia, Newborns, Associated factors, Ethiopia

## Abstract

**Background:**

Neonatal asphyxia is one of preventable causes of neonatal mortality throughout the world. It could be improved by early detection and control of the underlying causes. However, there was lack of evidence on it in the study setting. Thus, the aim of this study was to assess the magnitude and predictors of neonatal asphyxia among newborns at public hospitals of Wolaita Zone in Southern Ethiopia.

**Method:**

A facility-based cross-sectional study was done among 330 mothers with neonates in selected public hospitals. A systematic random sampling technique was used to select the study participants. Data were collected through an interviewer-administered questionnaire and checklist. The collected data were entered into EpiData version 4.6 and exported to SPSS version 26 for analysis. Logistic regression was fitted to examine the association between explanatory variables and outcome variable. In multivariable logistic regression, AOR with 95% CI was reported, and *p* < 0.05 was used to declare statistically significant variables.

**Results:**

The magnitude of neonatal asphyxia was 26.4% with 95% CI: (21.8, 30.9). In multivariable logistic regression analysis primiparity (AOR = 2.63 95%CI 1.47, 4.72), low-birth-weight (AOR = 3.45 95%CI 1.33, 8.91), preterm birth (AOR = 3.58 95%CI 1.29, 9.92), and premature rupture of membranes (AOR = 5.19 95%CI 2.03, 13.26) were factors significantly associated with neonatal asphyxia.

**Conclusions:**

In this study, the magnitude of neonatal asphyxia was high. From the factors, premature rapture of the membrane, parity, birth weight of the newborn, and gestational age at birth were significantly associated with neonatal asphyxia. Attention should be given to early detection and prevention of neonatal asphyxia from complicated labor and delivery.

## Introduction

One of the main causes of early newborn mortality is birth asphyxia, which is defined as the inability to establish breathing during birth and accounts for an estimated 900,000 deaths annually and obstetrical problems are the most frequent cause of it [1]. it is a main clinical problem that happens throughout the world and increases the risk of neonatal mortality and morbidity, and It is a condition which affects a newborn and results in decreased oxygen perfusion to various organs. It affects 2 to 10 cases per 1000 full-term babies; however it affects premature babies more frequent [2].

Globally, most of the neonatal mortality (75%) happens within the first week of the neonatal period and About 1 million newborns die within the first 24 h, and [3]*.* According to WHO statistics in developing country every year 120 million infants born, from this 3% develop birth asphyxia and it is responsible for 23% of newborn deaths in low-income countries, with sub-Saharan Africa accounting for 38% of these deaths [4–7].

Most cases of birth asphyxia are caused by disruptions in placental blood flow, which also affect fetal circulation. The placental vasculature may be impacted by maternal diseases like diabetes mellitus, hypertension, and pre-eclampsia, which can result in less blood flow. Fetal circulation is impacted by the effects of maternal hypotension caused on by medications, illnesses, and anesthesia [8]. The diagnosis of birth asphyxia is made when a newborn's Apgar score is less than seven and scores between zero and three indicate severe birth asphyxia, while those between four and seven indicate moderate birth asphyxia [9].

According to a comprehensive review, the incidence of asphyxia was 18% in East Africa and 22.52% in Ethiopia [10]. Additionally, according to 2019 Ethiopian Mini Demographic Health Survey (EDHS) report the neonatal mortality rate has slightly increased from 29 deaths per 1,000 live births in the 2016 EDHS report to 30 in 2019 [11], with birth asphyxia accounting for 13.5% of neonatal mortality cases [12], In developing countries, it continues to be a serious global clinical problem and about 29% of early neonatal deaths and 23.3% of all neonatal deaths were caused by it [13–15]. Ethiopia is one of the nations with the highest neonatal mortality in the world, with 30 deaths per 1000 live births [16]. Prenatal asphyxia is a significant cause of morbidity and mortality in Ethiopia, where it is exacerbated by a number of issues, such as low antenatal coverage (30% in Ethiopia), home deliveries, difficult access to healthcare facilities, a lack of qualified health workers, management faults, a lack of training in pediatric critical care, and inadequate transportation services [17]. Despite numerous initiatives to stop and decrease neonatal asphyxia, its prevalence was rising through time. Assessing the magnitude and identifying risk factors are important steps in reducing neonatal asphyxia related disorders and death. In addition, the finding from this study provides pertinent information for different planning and intervention programs of hospitals, and health bureaus in the study setting to reduce the neonatal mortality related to birth asphyxia Therefore, this study aimed to assess the magnitude and predictors of neonatal asphyxia in public hospitals of Wolaita Zone, Southern Ethiopia.

## Methods and materials

### Study design, area and period

A facility based cross- sectional study was conducted in a public hospitals of Wolaita Zone in Southern Ethiopia from May 1 to June 1, 2023, Which is located 394 km away from Addis Ababa, the capital city of Ethiopia, and 134 km far from Hawassa, the capital of the Southern Nations Nationalities People’s Regional state. Wolaita Zone has a total population of 5,385,282. The primary health coverage of Wolaita zone is provided according to the zonal health department report there are eight hospitals, out of this six governmental and two private hospitals; 69 health centers, and 24 private medium clinics and 342 health posts.

### Populations

The source population were all mothers having live birth neonates who delivered at gestational age ≥ 28 weeks in Wolaita zone, while the study population were all mothers having live birth neonates who delivered at gestational age ≥ 28 weeks in selected public hospitals of Wolaita zone during the data collection period.

### Eligibility criteria

We included newborns who delivered at a gestational age of 28 weeks and above during data collection period, whereas newborns with a major congenital malformation, congenital neuromuscular disorders, cardiovascular, central nervous system and pulmonary congenital disorders were excluded from the study.

### Sample size determination and sampling procedure

We calculated sample size by using a single population proportion formula with the assumptions; the margin of error (d) = 5%, confidence level = 95% (Zα/_2_ =  ± 1.96), and the proportion of neonatal asphyxia (p) was 27.1%, from a previous study conducted in South Gondar zone in Ethiopia [18]. So, the final calculated sample size with a 10% non-response rate was 335.

**Fig. 1:**
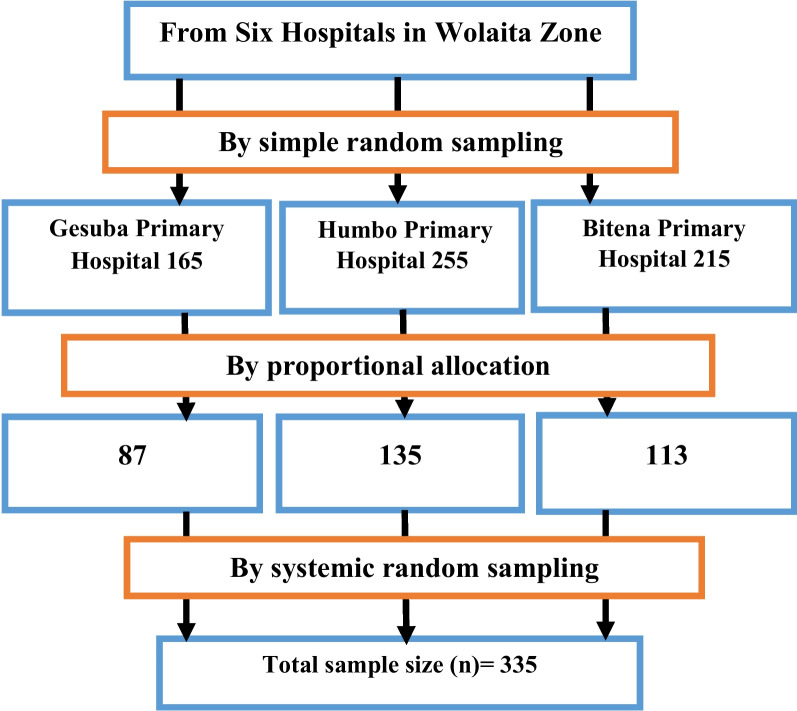
Schematic presentation of sampling procedure to assess the magnitude of neonatal asphyxia and its predictors in public hospitals of Wolaita Zone, Ethiopia 2023

Three public hospitals that provide NICU service were selected by simple random sampling technique from six public hospitals in the study area. Based on the monthly average number of deliveries, the sample were proportionally allocated to selected hospitals, Humbo Primary Hospital (135), Gesuba Primary Hospital (87) and Bitena Primary Hospital (113). Then, the study participants were selected by systematic random sampling technique at every K^th^ interval from each hospital and the first participant was selected by lottery method, as a starting point to select study participants from the sampling frame (Fig. 1).

### Study variables

The study’s explanatory variables include mother’s age, marital status, educational status, place of residency, occupation, anemia during pregnancy, antenatal care follow up, diabetes mellitus, preeclampsia/eclampsia, antepartum hemorrhage, chronic hypertension, gravidity, parity, premature rapture of membrane, prolonged labor, obstructed labor, and mode of delivery, birth weight, fetal distress, gender of neonate and presentation of the fetus.

### Data collection tools and procedures

An interviewer-based, semi-structured questionnaire was used to collect the data, along with a checklist for reviewing the medical records. The tools used to collect the data were adapted from previous relevant literature [19–22]. The questionnaire was prepared in English and translated to Amharic and back to English by a language expert to check its consistency.

The actual data collection was carried out from May 1 to June 1, 2023, by six trained bachelor degree midwives to record the data and three senior bachelor degree nurses to supervise the data quality. The data collection tools have four main parts: Maternal related factors, age, marital status, ethnicity, religion, residence, and occupational status), antepartum (parity, antepartum hemorrhage, co-existing obstetric/medical diseases, and antenatal visits), intra partum (duration of labor, fetal presentationmembranes), and neonatal related factors (asphyxia, gestational age, birth weight, sex, and birth type) were abstracted using a pre-tested structured questionnaire from the mothers who gave birth during the data collection period.

### Data quality control assurance

To maintain the data quality, the two-days training was given to data collectors and supervisors about the objective of the study, ethical issues, and data collection methods. The tool was translated into Amharic, and retranslated back to English to check the consistencies. Pre-test was done on 5% of the sample size in Boditi Primary Hospital before an actual data collection period to ensure the questionnaire was clear, error-free, and complete. Based on the results of the pretest, adjustments were made as needed, and the amended tool was used for an actual data collection. During the data collection period, close supervision and monitoring was carried out by supervisors and investigator to ensure the quality of the data. Daily evaluation of the data for completeness and encountered difficulties at the time of data collection was addressed accordingly. Finally, all the collected data were checked by the supervisor and investigator for its completeness, consistency, and clarity during the data management.

### Data processing and analysis

The collected data were cleaned, coded, and entered into EpiData version 4.6 software. Then, the data were exported to SPSS version 26 for analysis. Descriptive analysis was used to describe the characteristics of participants, and the result was described in the form of text, tables, and figures. By using a binary logistic regression model, bivariable analysis was done to identify the relationship between each independent variable and the outcome variable. Independent variables with *p* < 0.25 were included in the multivariable analysis to exclude potential confounders and identify actual associations. Multi-collinearity was checked to see the linear correlation among the independent variables by using the variance inflation factor and tolerance test. None of the variables yield variance inflation factor > 10 and tolerance test < 0.1. The model fitness was checked by the Hosmer Lemeshow goodness of fit test, it was found to be insignificant (*p* = 0.431), which indicates that the model was fitted. Then, in the multivariable logistic regression analysis, adjusted odds ratio (AOR) with a 95% confidence interval (CI) was reported, and variables with *p* < 0.05 were declared to be significantly associated with neonatal asphyxia.

### Operational definitions

#### Neonatal asphyxia

A newborn was considered to have neonatal asphyxia when its fifth minute APGAR score was less than seven [23, 24].

#### Prolonged labor

When duration of first stage of labor exceeds 14 h in multipara mothers and 20 h in primipara and second stage of labor 4 h and above in primipara mothers and 13 h and above in multipara [25].

#### Obstructed labor

Considered when the presenting part of the fetus could not progress into the birth canal, despite strong uterine contractions [26].

#### Premature rupture of membranes

Rupture of the membrane of the amniotic sac and chorion occurred before the onset of labor [27].

#### Gestational age

Is measured in weeks, from the first day of the woman's last menstrual cycle to the date of birth. Infants born before 37 weeks are considered as preterm, infants born 37 to 42 weeks was considered as term, Infants born after 42 weeks are considered as post term [28].

## Results

### Socio-demographic characteristics of the participants

In this study a total of 330 mothers of newborn were participated, with a response rate of 98.5%. The mean age of the respondents was 25.94 years with a standard deviation of ± 5.25 years. Of these, 122(37%) of mothers were found in the age group of 20 to 24 years and 152(46.1%) were urban dwellers. Regarding current marital status and occupation, 304(92.1%) were married and 207(62.7%) of respondents were housewives respectively. 236 (72.1%) of study participants completed secondary school or above (Table 1).

**Table 1:** Socio-demographic characteristics of study participants at public hospitals of Wolaita zone in southern Ethiopia, 2023. (*n* = 330)

Variable	Response	Frequency (n)	Percent (%)
Age in years	≤ 19	27	8.2
	20 – 24	122	37
	25 – 29	103	31.2
	30 – 34	44	13.3
	35 and above	34	10.3
Educational status	No formal education	6	1.8
	Primary school	86	26.1
	Secondary school	202	61.2
	College and above	36	10.9
Occupation	Government employee	38	11.5
	Private	35	10.6
	Merchant	34	10.3
	Housewife	207	62.7
	Student	16	4.8
Residence	Urban	152	46.1
	Rural	178	53.9
Marital status	Single	9	2.7
	Married	304	92.1
	Divorced	9	2.7
	Widowed	8	2.4

### Antenatal related factors

In this study, 320(97%) of the respondents had attended antenatal care follow up in the current pregnancy, even though, 84(25.5%) of them had less than four ANC visits. 209(63,3%) of respondents were multiparous and 270 (81.8%) of respondents gave birth at a term gestational age. About 30(9.1%) of mothers were anemic and 13(3.9%) have a preeclampsia/eclampsia. 10(3%) of mothers have antepartum hemorrhage and 3(0.9%) of respondents have gestational diabetes mellitus (Table 2).

**Table 2:** Antenatal Related Factors among the Participants in Wolaita Zone southern Ethiopia 2023 (*n* = 330)

Variables	Response	Frequency (n)	Percent (%)
Parity	Primipara	121	36.7
	Multipara	209	63.3
Have ANC follow-up	Yes	320	97
	No	10	3
Number of ANC visits	< 4 visits	84	25.5
	≥ 4 visits	246	74.5
Gravida	Primigravida	87	26.4
	Multigravida	243	73.6
Gestational age at birth	Preterm	36	10.9
	Term	270	81.8
	Post-term	24	7.3
Have anemia	Yes	30	9.1
	No	300	90.9
Preeclampsia/eclampsia	Yes	13	9.3
	No	317	96.1
APH	Yes	10	3
	No	320	97
Gestational DM	Yes	3	0.9
	No	327	99.1

Key: *APH* Antepartum hemorrhage, *DM* Diabetes mellitus, *ANC* Antenatal care visits

### Intrapartum related factors

In this study, 271(82.1%) of mothers were gave birth by spontaneous vaginal delivery, and 43(13%) of mothers had a prolonged labor, 294(89.1%) of newborn were delivered by vertex presentation, and 24(7.3%) of mothers were diagnosed for Premature rapture of membranes and only 6(1.8%) of mothers have an obstructed Labour (Table 3).

**Table 3:** Intrapartum Related Factors among the Participants in Wolaita Zone southern Ethiopia 2023 (*n* = 330)

Variables	Response	Frequency (n)	Percent (%)
Mode of delivery	Spontaneous vaginal	271	82.1
	Cesarean section	54	16.4
	Instrumental	5	1.5
Prolonged first stage of Labour	Yes	43	13
	No	287	87
Presentation of the fetus	Cephalic	294	89.1
	Non-cephalic	36	10.9
Diagnosed for PROM	Yes	24	7.3
	No	306	92.7
Obstructed labor	Yes	6	1.8
	No	324	98.2

Key: *PROM* Premature rapture of membranes

### Newborn related factors

Among the newborns included in this study, 173(52.4%) were female in sex and 262(79.4%) of newborns have a normal birth weight, while 73(22.1%) of newborns have a fetal distress during delivery and 264(80) of newborn were crying during delivery (Table 4).

**Table 4:** Newborn related factors among live birth who delivered at public hospitals of Wolaita Zone southern Ethiopia 2023 (*n* = 330)

Variables	Response	Frequency (n)	Percent (%)
Sex of the newborn	Male	153	46.4
	Female	173	52.4
Birth Weight	Low-birth weight	44	13.3
	Normal	262	79.4
	Macrosomia	24	7.3
Fetal distress at birth	Yes	73	22.1
	No	257	77.9

Key: *Kg* Kilograms

### Magnitude of neonatal asphyxia

**Fig. 2:**
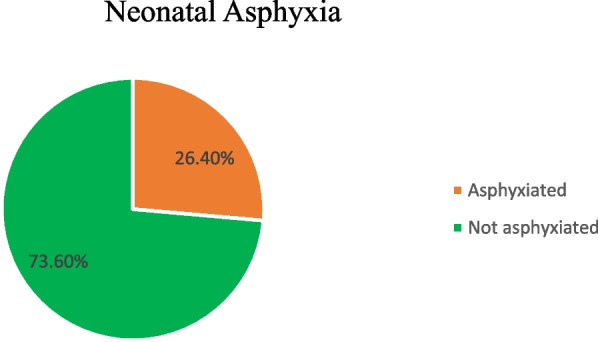
Neonatal Asphyxia among live births at public hospitals of Wolaita zone southern Ethiopia, 2023

The magnitude of neonatal asphyxia among live births at public hospitals of Wolaita zone in southern Ethiopia was found to be 26.4% with (95% CI: 21.8, 30.9) based on APGAR scoring less than seven at fifth minutes after birth (Fig. 2).

### Factors associated with neonatal asphyxia

In the bivariable logistic regression analysis, place of residence, parity, birth weight of the newborn, premature rapture of the membrane, gestational age at birth, maternal anemia, and sex of the newborns were identified as a candidate variable for multivariable analysis. However, after controlling for possible confounding factors on the multivariable logistic regression analysis, parity, birth weight of the newborn, premature rapture of the membrane, and gestational age at birth were factors significantly associated with neonatal asphyxia at 95% confidence level with *p* < 0.05.

The odds of neonatal asphyxia were 2.63 times higher among neonates born to Primiparous mothers than neonates born to multiparous mothers (AOR = 2.63 95%CI 1.47, 4.72). The odds of neonatal asphyxia were 3.45 times higher among low-birth-weight neonates than neonates with a normal birth weight (AOR = 3.45 95%CI 1.33, 8.91).

Furthermore, the odds of neonatal asphyxia were 3.58 times higher among preterm neonates than neonates delivered at a term gestational age (AOR = 3.58 95%CI 1.29, 9.92). In addition, neonates born to mothers with premature rupture of membranes were 5.19 times more likely to asphyxiated at birth as compared to those neonates born to mothers with no premature rupture of membranes (AOR = 5.19 95%CI 2.03, 13.26) (Table 5).

**Table 5:** Factors associated with neonatal asphyxia among live births at public hospitals of Wolaita zone southern Ethiopia, 2023 (*n* = 330)

Variables	Neonatal Asphyxia		COR (95% CI)	AOR (95% CI)	*p*-value
	**Yes (*n* = 87)**	**No (*n* = 243)**			
Place of residence					
Rural	62	116	2.71(1.60, 4.60)	1.59(0.86, 2.94)	0.139
Urban	25	127	1.00	1.00	
Parity					
Primiparous	50	71	3.27 (1.97, 5.43)	**2.63 (1.47, 4.72)**^a^	**0.001**
Multiparous	37	172	1.00	1.00	
Birth weight of the newborn					
< 2.5 kg	27	17	6.90 (3.49, 13.65)	**3.45 (1.33, 8.91)**^a^	**0.011**
2.5 to 4 kg	49	213	1.00	1.0	
> 4 kg	11	13	3.67 (0.68, 5.13)	1.33 (0.38, 4.67)	
Sex of the newborn					
Male	49	105	1.69 (1.03, 2.77)	0.95 (0.51, 1.74)	0.867
Female	38	138	1.00	1.00	
Gestational age at birth					
Preterm	24	12	8.38 (3.93, 17.85)	**3.58 (1.29, 9.92)**^a^	**0.014**
Term	52	218	1.00	1.00	
Post-term	11	13	2.36 (0.81, 6.82)	1.46 (0.40, 5.30)	0.558
Premature rupture of the membrane					
Yes	14	10	4.46 (1.90, 10.48)	**5.19 (2.03, 13.26)**^a^	**0.001**
No	73	233	1.00		
Maternal anemia					
Yes	18	12	5.02 (2.30, 10.93)	1.09 (0.36, 3.24)	0.875
No	69	231	1.00	1.00	

Key: *AOR* Adjusted odds ratio, *COR* Crude odds ratio, *CI* Confidence interval

^a^Shows significance

## Discussion

This study aimed to determine the magnitude of neonatal asphyxia and its associated factors among newborns at the public hospital in Wolaita Zone, Southern Ethiopia. The finding revealed that, the magnitude of neonatal asphyxia in this study setting was 26.4%. Among the factors like, parity, birth weight of the newborn, premature rapture of the membrane, and gestational age at birth were significantly associated with neonatal asphyxia.

The magnitude of neonatal asphyxia in this study setting was consistent with the finding of studies conducted in public hospitals of North Gondar Zone, Northwest Ethiopia (27.1%) [18], northeast Amhara, Ethiopia (22.6%) [29], in Tigray (22.1%) [19] and in Debre Tabor General Hospital, North Central Ethiopia (28.35%) [30].

However, this finding was lower than the result of studies done in Dilla Referral Hospital in Southern Ethiopia (32.8%) [20], Jimma zone public hospitals, Southwest Ethiopia (47.5%) [31] and in Iran (58.8%) [32]. This variation might be due to differences in sample size, study setting, and most crucially difference in case definition, since the done in Iran used additional laboratory-based results as diagnostic criteria for newborn asphyxia whereas our study only used the fifth minute APGAR score.

While, this study finding was higher than the results of other studies in the Public Health Facilities of Bahir Dar City, Northwest Ethiopia (21.7%) [33], Ayder Comprehensive Specialized Hospital, Northern Ethiopia (18%) [34] and in Harari and Dire Dawa Public Hospitals, eastern Ethiopia (20.8%) [35]. This discrepancy might be due to differences in sample size and study setting.

In this study, the odds of neonatal asphyxia were higher among neonates born to Primiparous mothers than neonates born to multiparous mothers. This finding is consistent with the result of studies conducted in Pakistan, Nigeria, and Tigray in Ethiopia [36, 37]. This consistency might be due to the fact that primipara mothers are more likely to give birth at a younger age, may be more prone for malpresentation, obstructed labor, and longer time to negotiate with the pelvic brim and may fail to progress in labor, resulting in the delivery of an asphyxiated neonate.

The odds of neonatal asphyxia were higher among low-birth-weight neonates than neonates with a normal birth weight. These finding was supported by the results of studies conducted in Jimma zone southwest Ethiopia [38], North Gondar Zone, Northwest Ethiopia [18], General hospitals in Gondar [38] and Iran [39]. This similarity might be due to the reason that many low birth weight newborns were preterm, they may have pulmonary immaturity and inadequate respiratory muscle strength and have breathing problems [40].

Furthermore, the odds of neonatal asphyxia were higher among preterm neonates than neonates delivered at a term gestational age. This finding was in line with a study conducted in Debre Markos Comprehensive Specialized Referral Hospital, Northwest Ethiopia [41], Ilu Aba Bor zone southwest, Ethiopia [42] and Bahir Dar City, Northwest Ethiopia [33]. The possible reason may be related to effects of preterm delivery, such as respiratory distress syndrome, which is brought on by immature lungs that are unable to maintain adequate oxygenation, leading to hypoxia, which damages the nervous system and results in conditions like cerebral palsy and necrotizing enterocolitis [43].

In addition, neonates born to mothers with premature rupture of membranes were more likely to asphyxiated at birth as compared to those neonates born to mothers with no premature rupture of membranes. This finding was consistent with the result of studies conducted in public hospitals of North Gondar Zone, Northwest Ethiopia [18], Mulago Hospital, Uganda [44], Saudi Arabia [45] and Urban Health Facility in Cameroon [46]. The possible reason might be due to oligohydramnios, the presenting part of the fetus presses on the umbilical cord and leads to umbilical cord compression, and obstruction of blood flow that carries maternal oxygen to the baby and causes birth asphyxia [47].

### Limitation of the study

The limitation of this study is that in our study we didn’t used laboratory-based results as diagnostic criteria for newborn asphyxia, in addition to this, cause and effect relationship between outcome and factors was not identified because of the nature of the cross-sectional study design.

## Conclusions

In this study area, the magnitude of neonatal asphyxia was high. The finding also identified that, from the factors parity, birth weight of the newborn, premature rapture of the membrane, and gestational age at birth were significantly associated factors with increased odds of neonatal asphyxia.

## Recommendations

Based on the findings of this study, the following recommendations were given:

### To health professionals

- ✓ Health care personnel, particularly those in labor and delivery wards, must pay more attention to complicated labors to foresee and prevent birth asphyxia, they also needed to strengthen their efforts to avoid complication during labor and delivery.

### To zonal health bureau

- ✓ The zonal health bureau should set up training services in neonatal asphyxia prevention.
- ✓ It is preferable to plan and deliver the tools and materials required for health professionals to advance their training on management of neonatal asphyxia.

### To researchers

- ✓ Additional follow up studies need to be conducted to address points that this study fails.

## Data Availability

The datasets used and/or analyzed during the current study are available from the corresponding author on reasonable request.

## Abbreviations

ANC: Antenatal Care
APGAR: Appearance, Pulse Rate, Grimace, Activity, Respiration Rate
APH: Antepartum Hemorrhage Care
GA: Gestational Age
LBW: Low Birth Weight
NICU: Neonatal Intensive Care Unit
NMR: Neonatal Mortality Rate
PROM: Premature Rupture of membrane
SNNPR: South Nations Nationalities People’s Region

## References

[1] Majeed R, Memon Y, Majeed F, Shaikh NP, Rajar UD (2007). Risk factors of birth asphyxia. J Ayub Med Coll Abbottabad.

[2] Antonucci R, Porcella A, Pilloni MD (2014). Perinatal asphyxia in the term newborn. J Pediatr Neonatal Individual Med (JPNIM).

[3] Organization WH. Newborns: reducing mortality, 2018. Retrieved 2020 Mar 11 from https://www.who.int/news-room/fact-sheets/detail/newborns-reducing-mortality. 2018.

[4] Organization WH. Guidelines on basic newborn resuscitation. 2012, from https://www.who.int/publications-detail-redirect/9789241503693. 23700652

[5] Lincetto O (2007). Birth asphyxia summary of the previous meeting and protocol overview.

[6] Wardlaw T, You D, Hug L, Amouzou A, Newby H (2014). UNICEF Report: enormous progress in child survival but greater focus on newborns urgently needed. Reprod Health.

[7] Gillam-Krakauer M, Gowen Jr CW. Birth asphyxia. 2017. 28613533

[8] Mir IN, Johnson-Welch SF, Nelson DB, Brown LS, Rosenfeld CR, Chalak LF (2015). Placental pathology is associated with severity of neonatal encephalopathy and adverse developmental outcomes following hypothermia. Am J Obstetr Gynecol.

[9] Thavarajah H, Flatley C, Kumar S (2018). The relationship between the five minute Apgar score, mode of birth and neonatal outcomes. J Matern Fetal Neonatal Med.

[10] Bayih WA, Birhane BM, Belay DM, Ayalew MY, Yitbarek GY, Workie HM (2021). The state of birth asphyxia in Ethiopia: an umbrella review of systematic review and meta-analysis reports, 2020. Heliyon.

[11] Lemma K, Misker D, Kassa M, Abdulkadir H, Otayto K (2022). Determinants of birth asphyxia among newborn live births in public hospitals of Gamo and Gofa zones, Southern Ethiopia. BMC Pediatr.

[12] Mersha A, Bante A, Shibiru S (2019). Neonatal mortality and its determinates in public hospitals of Gamo and Gofa zones, southern Ethiopia: prospective follow up study. BMC Pediatr.

[13] D'Alton ME, Hankins GD, Berkowitz RL, Bienstock J, Ghidini A, Goldsmith J, et al. Neonatal encephalopathy and neurologic outcome. Philadelphia: Lippincott Williams & Wilkins. 2014;896–901.

[14] Woday Tadesse A, Mekuria Negussie Y, Aychiluhm SB (2021). Neonatal mortality and its associated factors among neonates admitted at public hospitals, pastoral region, Ethiopia: a health facility based study. Plos One.

[15] Ayebare E, Hanson C, Nankunda J, Hjelmstedt A, Nantanda R, Jonas W (2022). Factors associated with birth asphyxia among term singleton births at two referral hospitals in Northern Uganda: a cross sectional study. BMC Pregnancy Childb.

[16] Sharrow D, Hug L, You D, Alkema L, Black R, Cousens S (2022). Global, regional, and national trends in under-5 mortality between 1990 and 2019 with scenario-based projections until 2030: a systematic analysis by the UN Inter-agency group for child mortality estimation. Lancet Glob Health.

[17] Kali GTJ, Martinez-Biarge M, Van Zyl J, Smith J, Rutherford M (2015). Management of therapeutic hypothermia for neonatal hypoxic ischaemic encephalopathy in a tertiary centre in South Africa. Arch Dis Child Fetal Neonatal Ed.

[18] Admasu FT, Melese BD, Amare TJ, Zewude EA, Denku CY, Dejenie TA (2022). The magnitude of neonatal asphyxia and its associated factors among newborns in public hospitals of North Gondar Zone, Northwest Ethiopia: a cross-sectional study. Plos One.

[19] Gebreheat G, Tsegay T, Kiros D, Teame H, Etsay N, Welu G (2018). Prevalence and associated factors of perinatal asphyxia among neonates in general hospitals of Tigray Ethiopia 2018. BioMed Res Int.

[20] Alemu A, Melaku G, Abera GB, Damte A (2019). Prevalence and associated factors of perinatal asphyxia among newborns in Dilla University referral hospital, Southern Ethiopia–2017. Pediatr Health, Med Ther.

[21] Ibrahim N, Muhye A, Abdulie S (2017). Prevalence of birth asphyxia and associated factors among neonates delivered in Dilchora Referral Hospital. Dire Dawa, Eastern Ethiopia Clin Mother Child Health.

[22] Ndombo PK, Ekei QM, Tochie JN, Temgoua MN, Angong FTE, Ntock FN (2017). A cohort analysis of neonatal hospital mortality rate and predictors of neonatal mortality in a sub-urban hospital of Cameroon. Ital J Pediatr.

[23] Bayih WA, Tezera TG, Alemu AY, Belay DM, Hailemeskel HS, Ayalew MY (2021). Prevalence and determinants of asphyxia neonatorum among live births at Debre Tabor General Hospital, North Central Ethiopia: a cross-sectional study. Afr Health Sci.

[24] Federal democratic republic of Ethiopia Ministry of Health BL. Best practice in maternal and newborn care Maternal Death Surveillance and Response. 2018.

[25] LeFevre NM, Krumm E, Cobb WJ (2021). Labor Dystocia in Nulliparous patients. Am Fam Phys.

[26] Bako B, Barka E, Kullima AA (2018). Prevalence, risk factors, and outcomes of obstructed labor at the University of Maiduguri teaching hospital, Maiduguri, Nigeria. Sahel Med J.

[27] Mulugeta T, Sebsibe G, Fenta FA, Sibhat M (2020). Risk factors of perinatal asphyxia among newborns delivered at public hospitals in Addis Ababa, Ethiopia: case–control study. Pediatr Health, Med Ther.

[28] Yerushalmy J (1967). The classification of newborn infants by birth weight and gestational age. J Pediatr.

[29] Abubakari A, Kynast-Wolf G, Jahn A (2015). Prevalence of abnormal birth weight and related factors in Northern region Ghana. BMC Pregn Childb.

[30] Woday A, Muluneh A, St DC (2019). Birth asphyxia and its associated factors among newborns in public hospital, northeast Amhara, Ethiopia. Plos One.

[31] Bayih WA, Yitbarek GY, Aynalem YA, Abate BB, Tesfaw A, Ayalew MY (2020). Prevalence and associated factors of birth asphyxia among live births at Debre Tabor General Hospital, North Central Ethiopia. BMC Pregnancy Childb.

[32] Wayessa ZJ, Belachew T, Joseph J. Birth asphyxia and associated factors among newborns delivered in Jimma zone public hospitals, Southwest Ethiopia: a cross-sectional study. J Midw Reprod Health 2018;6(2):1–9.

[33] Nayeri F, Shariat M, Dalili H, Adam LB, Mehrjerdi FZ, Shakeri A (2012). Perinatal risk factors for neonatal asphyxia in Vali-e-Asr hospital, Tehran-Iran. Iranian J Reprod Med.

[34] Dabalo ML, AnimenBante S, Belay Gela G, Lake Fanta S, AbdisaSori L, FeyisaBalcha W (2021). Perinatal asphyxia and its associated factors among live births in the public health facilities of Bahir Dar City, Northwest Ethiopia, 2021. Int J Pediatr.

[35] Gebregziabher GT, Hadgu FB, Abebe HT. Research article prevalence and associated factors of perinatal asphyxia in neonates admitted to ayder comprehensive specialized hospital, Northern Ethiopia: a cross-sectional study. 2020. 10.1155/2020/4367248 PMC7042545 32110243

[36] Amsalu S, Dheresa M, Dessie Y, Eshetu B, Balis B (2023). Birth asphyxia, determinants, and its management among neonates admitted to NICU in Harari and Dire Dawa Public Hospitals, eastern Ethiopia. Front Pediatr.

[37] Prasad P, Shruti S. Antenatal and intrapartum risk factors for perinatal asphyxia: A case con-trol study.

[38] Gebremedhin M, Ambaw F, Admassu E, Berhane H (2015). Maternal associated factors of low birth weight: a hospital based cross-sectional mixed study in Tigray Northern Ethiopia. BMC Pregn Childb.

[39] Wosenu L, Worku AG, Teshome DF, Gelagay AA (2018). Determinants of birth asphyxia among live birth newborns in University of Gondar referral hospital, northwest Ethiopia: a case-control study. Plos One.

[40] Jagkaew B (2014). Factors associated with birth asphyxia in Fang Hospital. Mahasarakham Hosp J.

[41] Speer CP (2011). Neonatal respiratory distress syndrome: an inflammatory disease?. Neonatology.

[42] Alamneh YM, Negesse A, Aynalem YA, Shiferaw WS, Gedefew M, Tilahun M (2022). Risk factors of birth asphyxia among newborns at Debre Markos comprehensive specialized referral hospital, Northwest Ethiopia: unmatched case-control study. Ethiop J Health Sci.

[43] Fekede T, Fufa A (2022). Determinants of birth asphyxia at public hospitals in Ilu Aba Bor zone southwest, Ethiopia: a case control study. Sci Rep.

[44] Dashe JS, Bloom SL, Spong CY, Hoffman BL. Williams obstetrics. United States: McGraw Hill Professional; 2018.

[45] Kaye D (2003). Antenatal and intrapartum risk factors for birth asphyxia among emergency obstetric referrals in Mulago Hospital, Kampala Uganda. East Afr Med J.

[46] Sahib HS (2015). Risk factors of perinatal asphyxia: a study at Al-Diwaniya maternity and children teaching hospital. Risk.

[47] Chiabi A, Nguefack S, Evelyne M, Nodem S, Mbuagbaw L, Mbonda E (2013). Risk factors for birth asphyxia in an urban health facility in Cameroon. Iran J Child Neurol.

